# Assessment of the Biochemical Responses of Wheat Seedlings to Soil Drought after Application of Selective Herbicide

**DOI:** 10.3390/plants10040733

**Published:** 2021-04-09

**Authors:** Dessislava Todorova, Iskren Sergiev, Zornitsa Katerova, Elena Shopova, Ljudmila Dimitrova, Liliana Brankova

**Affiliations:** Institute of Plant Physiology and Genetics—Bulgarian Academy of Sciences, Acad G. Bonchev Str., Bl. 21. 1113 Sofia, Bulgaria; dessita@bio21.bas.bg (D.T.); zkaterova.landzhova@gmail.com (Z.K.); kostei@abv.bg (E.S.); dim.lyudmila@gmail.com (L.D.); lbrankova@abv.bg (L.B.)

**Keywords:** antioxidants, drought, herbicide, stress markers, wheat

## Abstract

Drought is a major environmental constrain with a deleterious effect on plant development leading to a considerable reduction of crop productivity worldwide. Wheat is a relatively drought tolerant crop during the vegetative stage. The herbicide Serrate^®^ (Syngenta) is a preparation containing two active chemical substances with different modes of action, which inhibit the biosynthesis of fatty and amino acids. It is commonly used as a systemic and selective chemical agent to control annual grass and broadleaf weeds in cereal crops and particularly in wheat, which is tolerant to Serrate^®^. Seventeen-day-old wheat seedlings (*Triticum aestivum* L., cv. Sadovo-1) grown as soil culture under controlled conditions were sprayed with an aqueous solution of Serrate^®^. Seventy-two hours later the plantlets were subjected to drought stress for seven days to reach a severe water deficit followed by four days of recovery with a normal irrigation regime. Oxidative stress markers, non-enzymatic, and enzymatic antioxidants were analyzed in the leaves of plants from the different treatment groups (herbicide-treated, droughts-stressed, and individuals which were consecutively subjected to both treatments) at 0, 96, and 168 h of drought stress, and after 96 h of recovery. Herbicide treatment did not alter substantially the phenotype and growth parameters of the above-ground plant parts. It provoked a moderate increase in phenolics, thiol-containing compounds, catalase, superoxide dismutase, glutathione reductase, and H_2_O_2_. However, significant variations of malondialdehyde, proline, and peroxidase activity caused by the sole application of the herbicide were not detected during the experimental period. Drought and herbicide + drought treatments caused significant growth inhibition, increased oxidative stress markers, and activation of enzymatic and non-enzymatic antioxidant defense reaching the highest levels at 168 h of stress. Plant growth was restored after 96 h of recovery and the levels of the monitored biochemical parameters showed a substantial decline. The herbicide provoked an extra load of oxidative stress-related biochemical components which did not aggravate the phenotypic and growth traits of plants subjected to drought, since they exhibited a good physiological status upon recovery.

## 1. Introduction

During the last decades, human activity and the unwise use of natural resources has contributed to the continually deepening adverse climate changes on Earth. NASA reports [1] show that since the late twentieth century the average temperature has increased by 0.8 °C, which along with the increasing frequency of climatic anomalies such as periods of heavy drought or torrential rainfalls cause significant losses in the yield of important crops. Based on a number of climate models, it is predicted that in many regions the environmental changes will deepen and lead to even more tangible decline in plant productivity [2].

Drought is one of the environmental factors affecting almost all aspects of plant development. Physiological drought in plants can occur due to water scarcity or soil water unavailability, soil salinity, and elevated air temperature [3]. Water deficiency adversely affects the germination, growth, and reproduction of plants. This stressor is directly linked to decrease of crop productivity since it disrupts major biochemical and physiological processes.

Weeds present another important limiting factor for crop breeding. The discovery of chemical substances selectively destroying weeds benefits crop growth and insures high crop yields. In this respect, herbicides as plant protection products against weeds are an integral part of the modern agriculture. Herbicidal active compounds can be separated into four general groups according to their mechanism of action: herbicides affecting photosynthesis or photosynthetic pigments; auxin-type herbicides; inhibitors of amino acid biosynthesis, and inhibitors of fatty acid biosynthesis. According to the sensitivity of plants to herbicidal action, herbicides are classified as selective, affecting certain types of plant species, and total, affecting all plant species.

Serrate^®^ (Syngenta, Bazel, Switzerland) is a herbicide consisting of two active substances: clodinafop-propargyl (prop-2-ynyl(*R*)-2-[4-(5-chloro-3-fluoro-2-pyridyloxy)phenoxy] propionate) inhibitor of acetyl co-enzyme A carboxylase, which is essential for the fatty acids biosynthesis; and pyroxsulam ([N-(5,7-dimethoxy [1,2,4]triazolo [1,5-a]pyrimidin-2 -yl)-2-methoxy-4-(trifluoromethyl)pyridine-3-sulfonamide]) inhibitor of acetolactate synthase enzyme, which catalyzes a key step in the biosynthesis of the branched-chain amino acids. The preparation contains also a safener and is highly effective in controlling annual grass and broadleaf weeds in cereal crops. The herbicide is systemic and is selective for wheat, rye, and triticale, which all are tolerant to the preparation.

The cultivation of plants under sub-optimal conditions is accompanied by unfavorable oxidative events in the cells, whereby reactive oxygen species (ROS) are formed as a universal physiological response to various types of abiotic stress factors, including drought. Accumulation of free radicals can induce chain oxidation reactions leading to the formation of lipid peroxides, which damage the biomembranes [4,5]. This disrupts fundamental physiological processes and adversely affects the physiological status of the plant organism. Numerous publications investigate the effect of a single stress factor (low or high temperature, salinization, radiation, herbicides, water or mineral deficiency, heavy metals, etc.) on the physiological status of the plant and/or the possibility of reducing the impact of the stress [6,7,8,9,10]. Under natural conditions, however, plants are rarely exposed to a single stress factor. Usually, plants are subjected to the simultaneous influence of several stressors. Often their additive damaging impact exceeds by far the effect of their self-administration, a phenomenon called cross-synergism [11,12]. Such an additive damaging effect has been observed in atrazine-treated maize grown under high soil moisture and low temperatures [13]. Exposure of drought-stressed wheat or tobacco plants to additional high-temperature had a stronger negative impact compared to the alone-applied drought [14,15,16].

Previously there have been published results describing studies in which plants subjected to a single stress agent exhibited improved tolerance to the subsequent adverse environmental conditions (cross-adaptation). For example, cross-adaptation has been reported in rice subjected to PEG and LiCl [17]; cucumber seedlings after combined heat and salinization [18]; grapes after drought combined with UV-B radiation [19]. Increased resistance has been observed in drought-stressed pea plants treated with high temperature [20]. An increase in salinity resistance has been observed in some medicinal plants preliminary irradiated with UV-B, and a similar effect was found also after NaCl pretreatment and subsequent UV-B irradiation [21]. Cross-adaptation has been also documented in barley treated with different heavy metals [22]. Many ambiguous points of discussion regarding the interaction between two and/or more stressors still exist and there are a number of gaps related to the understanding of the mechanisms by which cross-adaptation and cross-synergism operate.

Plants treated with herbicides, similarly to those grown under various abiotic stress conditions, are subjected to enhanced attacks by Reactive Oxygen Species (ROS) [23,24]. Plants have developed complex endogenous hormonal, enzymatic and non-enzymatic protective systems to overcome the adverse effects of ROS that have evolved during their evolution [4]. These mechanisms are implicated also in the processing or neutralization of herbicides by converting them to non-toxic metabolites [25]. The use of herbicides is usually not recommended on crops subjected to stress such as flooding, frost, drought, nutritional deficiency, etc., and often it is explicitly stated in the instructions for application provided by the manufacturers. However, there is no information on how subsequent adverse environmental conditions could affect the physiological status of herbicide-treated plants, which often occurs in the field. The question whether and how the herbicide will affect the physiological responses of plants when exposed to the effects of a subsequent abiotic stress factor remain unclear. Will the herbicide application cause a cross-synergism resulting in exaggerated negative effects on the physiological status of the treated plants, or will it possibly improve their adaptive capacity and stress tolerance? The study addresses this yet unresolved question by evaluating the physiological responses of wheat seedlings to soil drought after application of the selective herbicide Serrate^®^.

## 2. Results

### 2.1. Growth Parameters

**Figure 1:**
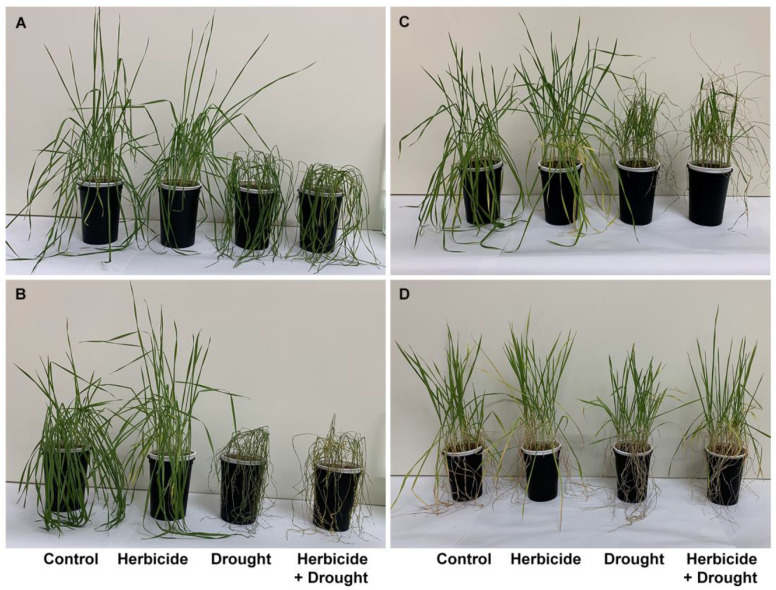
Phenotypic traits of wheat plants treated with herbicide and subjected to drought stress. (**A**) 96 h of drought, (**B**) 168 h of drought, (**C**) 96 h of recovery after restoring the normal irrigation regime, (**D**) 24 days of recovery.

**Figure 2:**
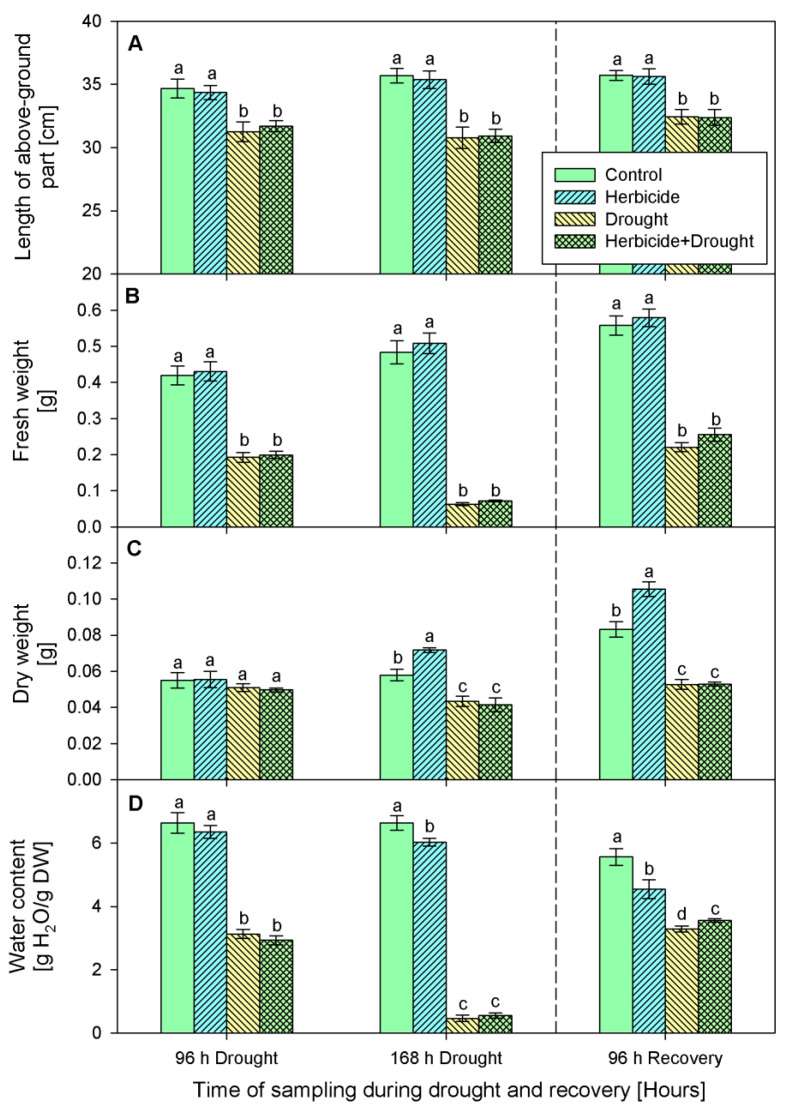
Length (**A**), fresh weight (**B**), dry weight (**C**), water content (**D**) of above-ground part of wheat treated with herbicide and exposed to drought. Control values at 0 h: (**A**) 32.1 ± 0.4 cm; (**B**) 0.331 ± 0.011 g; (**C**) 0.041 ± 0.001 g; (**D**) 6.922 ± 0.061 g. The same letter means lack of statistical significance between treatments (*n* = 3, *p* < 0.05).

The changes of crop phenotypic and growth traits due to drought stress and herbicide treatment are presented in Figure 1 and Figure 2. Apparently, the herbicide application did not provoke significant alterations in plant growth and fresh biomass. However, it affected the dry weight which increased by 24% at 168 h of drought and by 27% at 96 h of recovery. This was linked to reduced water content (by 9% at 168 h of drought and by 18% at 96 h of recovery) and yellowing of the older leaves. The alterations caused by drought and drought + herbicide treatments were similar and depended on drought duration. Significant wilting (Figure 1A) and inhibition of plant elongation growth (by 10%), fresh weight (by 54%), and water content (by 56%) at 96 h of stress was observed (Figure 2).

These negative effects were stronger at 168 h of drought stress with 18% growth inhibition, reduction of fresh weight by 87%, and water loss by 93% (Figure 1B and Figure 2). A reduction in dry weight (by 28%) was observed also. After restoring the irrigation, the drought-stressed plants resumed their growth (Figure 1C) and the plants acquired normal status comparable to the controls after 24 days of recovery (Figure 1D). The measured water content reached approximately 59% of the control level in drought-treated and 64% in combine-treated at 96 h of recovery (Figure 2D). Accordingly, fresh weight increased up to 40% in drought-treated and up to 45% in combine-treated plants as compared to the control (Figure 2B). Length of above-ground part and dry weight were not significantly altered and remained lower than the respective controls (Figure 2A,C).

### 2.2. Stress Markers Content

**Figure 3:**
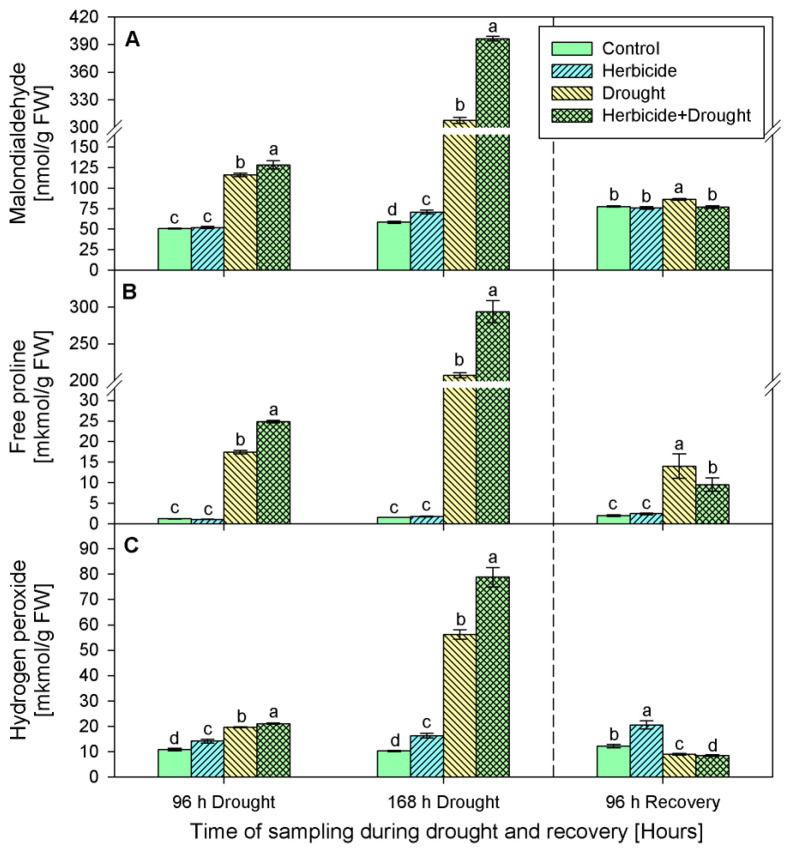
Content of malondialdehyde (**A**), free proline (**B**), and hydrogen peroxide (**C**) in leaves of wheat treated with herbicide and exposed to drought. Control values at 0 h: (**A**) 57.210 ± 4.104; (**B**) 0.837 ± 0.039; (**C**) 6.568 ± 0.696. The same letter means lack of statistical significance between treatments (*n* = 3, *p* < 0.05).

The changes in malonedialdehyde (MDA) content of plants treated only with herbicide during the first 96 h of drought and after recovery were insignificant compared to the respective control (Figure 3A). At 168 h a slight increase by 20% was detected. MDA accumulation was the most prominent in both drought stressed variants—drought only and drought in combination with herbicide. After 96 h of stress MDA content increased significantly by 129% (drought stressed) and by 153% (herbicide treatment followed by drought). Later, at 168 h of stress, its amount drastically raised reaching 424% and 576% respectively. After 96 h of recovery MDA quantity dropped to the control levels and remained slightly increased by 11% only in the drought-stressed seedlings.

Similar alterations were found in proline content (Figure 3B). During the stress and recovery periods proline level in herbicide treated plants varied insignificantly as compared to the respective controls. However, drought stress provoked considerable increase in proline content as follows: 15-fold (after 96 h of drought), 134-fold (after 168 h of drought), and 7-fold (after 96 h of recovery) in drought treated seedlings and 21-fold, 190-fold, and 5-fold, respectively, in the plants subjected to the combined herbicide+drought treatment.

Hydrogen peroxide (Figure 3C) was continuously accumulated in the herbicide treatment group by 31% (96 h of drought), 58% (168 h of drought), and 70% (96 h of recovery). Drought caused an increase of H_2_O_2_ by 82% (96 h of drought) and by 446% (168 h of drought). Upon recovery H_2_O_2_ content was below the control levels by 26%. The combined treatment provoked more prominent changes in this parameter — initially, at 96 h of drought, H_2_O_2_ was augmented 2-fold, followed by a 7.5-fold increase (168 h of drought) and sharp drop after recovery which was 30% below the control levels.

### 2.3. Activity of Antioxidant Enzymes

**Figure 4:**
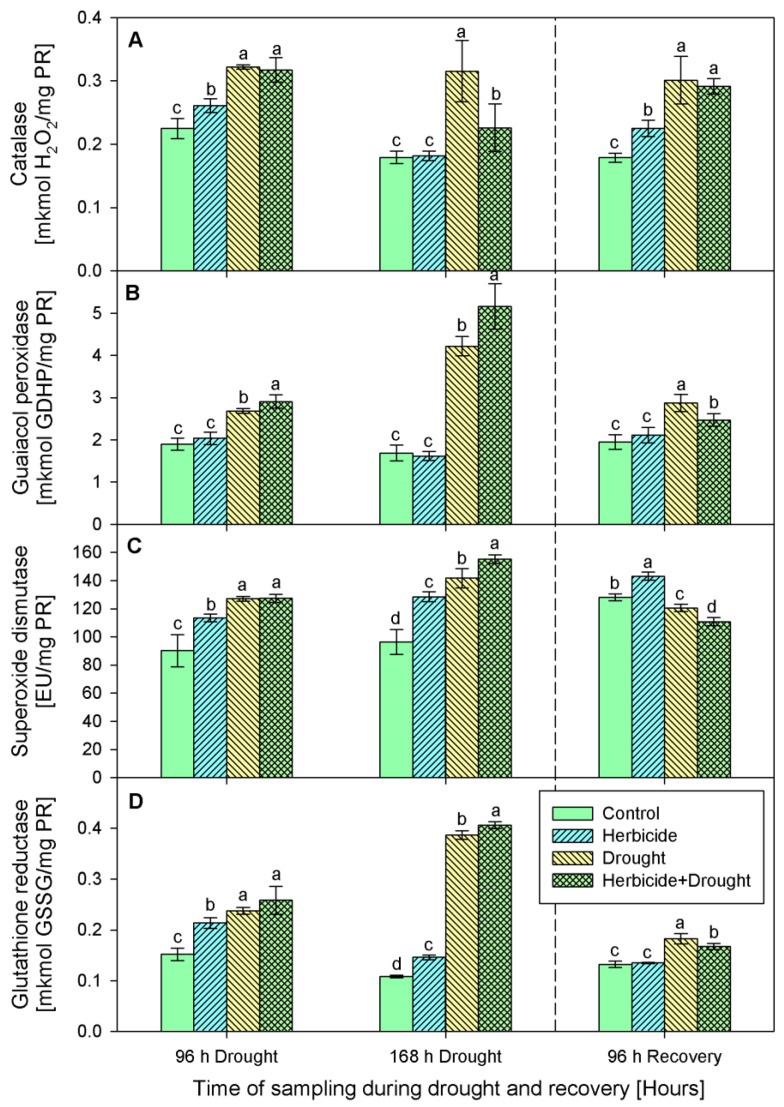
Activity of catalase (**A**), guaiacol peroxidase (**B**), superoxide dismutase (**C**), and glutathione reductase (**D**) in leaves of wheat treated with herbicide and exposed to drought. Control values at 0 h: (**A**) 0.211 ± 0.020; (**B**) 1.767 ± 0.192; (**C**) 60.280 ± 9.942; (**D**) 0.158 ± 0.012. The same letter means lack of statistical significance between treatments (*n* = 3, *p* < 0.05).

Increased catalase activity (Figure 4A) was found in drought and drought-herbicide treated seedlings during the whole experimental period. The highest levels of enzymatic activity were detected during the first 96 h of drought, then after the recovery catalase tended to decrease, but remained higher than the controls. The catalase activity changes provoked by the herbicide alone were less prominent.

Guaiacol peroxidase activity (Figure 4B) was not changed significantly by the herbicide as compared to the respective control levels at each sampling point of the experimental period. Drought stress applied in both variants—alone and in combination with the herbicide provoked a noticeable increase of the guaiacol peroxidase activity and it reached 181% at 168 h of drought in the herbicide+drought-treated seedlings. Elevated SOD (Figure 4C) was detected during the first 168 h of stress in all experimental groups. Upon recovery, the activity dropped below the control levels except in the plants treated only with herbicide where it remained above the controls by 11%. Glutathione reductase (GR) activity (Figure 4D) was consistently induced by all the treatments during the stress period, and the highest activity was detected in drought-stressed and herbicide+drought-treated seedlings—up to fourfold as compared to the control level. GR activity returned to the control levels in herbicide-treated seedlings after 96 h of recovery, while in the drought-stressed and drought+herbicide-treated group it remained slightly higher.

### 2.4. Content of Non-Enzymatic Antioxidants

**Figure 5:**
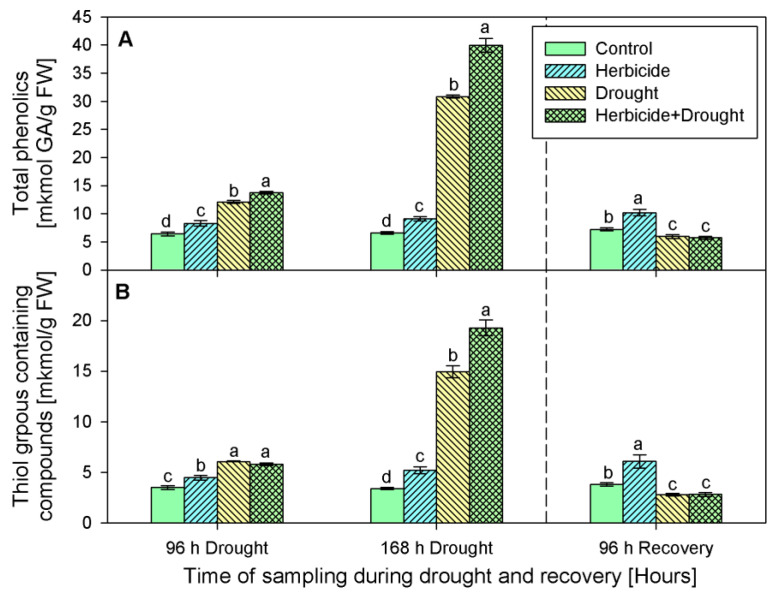
Content of total phenolics (**A**), and thiol groups containing compounds (**B**) in leaves of wheat treated with herbicide and exposed to drought. Control values at 0 h: (**A**) 4.557 ± 0.223; (**B**) 2.198 ± 0.115. The same letter means lack of statistical significance between treatments (*n* = 3, *p* < 0.05).

Phenolics content progressively increased after the herbicide treatment by 29% (at 96 h-stress sampling point), by 38% (at 168 h of the stress program), and by 41%, measured at the recovery sampling point (Figure 5A). A similar trend was observed in the content of thiol-containing compounds (Figure 5B). Their amounts continuously increased in the samples derived from herbicide-only treated plants as follows: by 28% (at 96 h), 52% (at 168 h), and 60% (after recovery).

Drought, alone or in combination with the herbicide, also caused an increase in the levels of these antioxidants. Phenolics raised by 89% and 367% in drought-stressed seedlings, and by 115% and 506% in the herbicide+drought combination-treated plants (Figure 5A). Phenolics content dropped below the control (by 20%) upon recovery. Significant differences in thiol-containing compounds (-SH) between both drought-stressed variants during the first 96 h of stress were not detected. An increase in their quantity by approximately 70% was found in both experimental groups at this sampling point (i.e., 96 h of drought). A peak of -SH content was found after 168 h of stress — drought alone led to an increase by 338%, and the herbicide treatment caused an additional increase with up to 465% under drought stress conditions. The measured thiol-containing compounds upon recovery were lower than the control by 27% (Figure 5B).

## 3. Discussion

As an environmental stress factor, drought disrupts plant metabolism and physiological responses. Drought stress elicits excessive formation of ROS, such as O_2_^•−^ and H_2_O_2_ in plant cells, which provokes injury of cellular membranes due to peroxidation of membrane phospholipids, which causes accumulation of MDA [26]. To scavenge the extra generated ROS, plants activate an efficient antioxidant enzymatic and non-enzymatic defense systems [26,27]. Enzymatic antioxidants include enzymes like superoxide dismutase (SOD), catalase (CAT), ascorbate peroxidase (APX), glutathione peroxidase (POD), and glutathione reductase (GR). SOD acts as a front line shield converting O_2_^•−^ to H_2_O_2_. Then CAT, APX, and POD detoxify H_2_O_2_ forming H_2_O. Non-enzymatic antioxidants including glutathione (GSH), ascorbic acid (AsA), carotenoids, tocopherols, and some phenolics like flavonoids are also involved in maintaining ROS homeostasis in plants [26,27]. Wheat is particularly vulnerable to drought at the flowering and grain development stages, but it can tolerate mild and moderate drought during vegetation [28], thus young drought-treated plants are capable to recover after the normal water supply is restored [29,30] as it was confirmed in our experiments (Figure 1 and Figure 2). Wheat also tolerates some selective herbicides like Serrate^®^ as evident by the lack of growth inhibition in herbicide-treated plants (Figure 2). Our results present biochemical responses to treatment with Serrate^®^ and subsequent stress during the early vegetative stage in winter wheat, information which to our knowledge was missing so far. To fill in this gap we assessed the levels of some stress markers, non-enzymatic, and enzymatic antioxidants in treated with herbicide and drought wheat seedlings during the stress and recovery phases.

We detected a significant boost of stress markers MDA, H_2_O_2_, and proline in drought-stressed plants (Figure 3) which is in line with the well-established fact that water deprivation induces generation of ROS, which can diffuse across the cell biomembranes and cause cell damage. Similar increase in the stress markers content was documented previously in wheat [31,32,33] and tobacco [16] drought-stressed plants. Along with the increase in the stress markers, a significant induction of antioxidant enzymes, non-enzymatic antioxidants, and compatible solutes in wheat was reported by Abid et al. [33], Sallam et al. [34] and the references therein. An activation of plant defense systems was observed in the present study as well and this was manifested by the substantial progressive increase of enzymatic and non-enzymatic antioxidants (Figure 4 and Figure 5). Similar patterns have been reported by other authors [16,33,35,36] in other model systems. Usually, when a certain environmental constrain is no longer present, plant metabolism enters into a recovery regime that tends to bring the physiological parameters in the plant system to their initial levels [31,35,37]. Accordingly, we found that after restoring the irrigation, there was a significant decline in values of the measured traits (Figure 3, Figure 4 and Figure 5) as compared to those detected at 168 h of drought, except for the catalase activity. This drop indicated that upon recovery, plants were capable to regain normal physiological status. As it was reported earlier, re-watering of drought-stressed plants restored partially the plant metabolism in tobacco [16] and wheat [31,33].

Limited number of articles reported the alterations in some agronomic, physiological and/or biochemical traits after individual application of clodinafop-propargyl [38,39,40,41] and pyroxsulam [42,43]. Our study communicates the results describing the biochemical alterations in wheat after application of Serrate^®^, which contains both active ingredients clodinafop-propargyl and pyroxsulam. We observed that the herbicide alone did not cause significant changes in most of the studied parameters, including the monitored stress markers (MDA and proline), which suggests that the herbicide treatment does not result in strong oxidative stress events. This is in line with the characteristics of the preparation provided by the manufacturer stating that wheat is tolerant to Serrate^®^ application. Relatively higher values of H_2_O_2_, non-enzymatic antioxidants, and SOD activity were found in herbicide-treated plants, which remained steadily elevated throughout the experimental period. Temporary increases were also observed in GR and catalase activity. Moderately high levels of H_2_O_2_ seem to function as a signal that triggers protective responses [44,45]. In accordance with this, our results suggest that higher herbicide-induced levels of H_2_O_2_ might trigger antioxidant protection judging by the increased quantity of non-enzymatic antioxidants and SOD activity measured in the herbicide-treated plants long after the application as evident from the measurements taken at the recovery sampling point. Simultaneous or subsequent occurrence of more than one unfavorable circumstances can influence either positively or negatively the plants physiological status [46]. The positive physiological reactions are characterized as cross-adaptation or cross-tolerance that induce adaptive response rendering tolerance to a follow-up stressor. This phenomenon is known as priming and it has been related to a more efficient activation of the plant defense responses [45,47,48,49]. The results of priming may last for several days via induction of the so-called stress memory [45,47,48]. The stress memory enables plants to be more tolerant to future environmental constraints [47] and it can be triggered by mild biotic or abiotic stressors, by beneficial microbes, as well as by chemical priming through the application of natural and synthetic compounds [27,48,49]. In addition some plant protection chemicals as herbicides and fungicides are able to act as cross-tolerance elicitors [48,49]. In most cases, during and after priming, plants generate ROS, especially H_2_O_2_ that can act as early response signaling molecule to boost several antioxidant mechanisms, which plants memorize [45]. This facilitates the more rapid and efficient plant response to the subsequent stress.

On the opposite, cross-synergism may occur as an additive effect of negative consequences of two or more adverse factors [14,15,32]. We found that combined treatment caused more dramatic alteration in biochemical traits during the stress period as compared to drought treatment alone. The levels of stress markers (Figure 3) and antioxidant defense machinery (Figure 4 and Figure 5) were higher in herbicide+drought combination-treated plants than those in drought-stressed only. Nonetheless the drastically different levels of physiological responses during the stress period, upon restored irrigation the biochemical parameters tended to recover to a similar state.

We suggest that herbicide application did not provoke a typical cross-synergistic response in the drought-stressed plants since they did not exhibit worsen phenotypic and growth traits, and they recovered successfully after re-watering, particularly evident after a prolonged recovery period (Figure 1D).

## 4. Materials and Methods

### 4.1. Plant Material and Treatments

Wheat (*Triticum aestivum* L., cv. Sadovo-1) was obtained from the Institute of Plant Genetic Resources (Sadovo, Bulgaria). The plants were grown under controlled conditions (22 °C/17 °C (day/night) temperatures, 16/8 h (day/night) photoperiod, and 60% relative air humidity) on pots filled with leached meadow cinnamon soil (pH 6.2) delivered from the Institute’s experimental field near Sofia. Each pot consisted of 20 plants. Seventeen-day-old seedlings (2–3 leaf phase) were treated with aqueous solution of the herbicide Serrate^®^ according to manufacturer’s instructions (25 g/d). After 72 h part of the plants were exposed to drought stress by withholding watering for 7 days to reach water deficit of 60% below the normally irrigated control variants [29,31,50]. Then the normal irrigation was restored, and the stressed plants were left to recover for 4 days.

The plants were divided into the following groups:

1. Normally irrigated control plants.
2. Normally irrigated plants treated with herbicide.
3. Drought stressed plants.
4. Plants treated with herbicide and subsequently exposed to drought stress.

Samples were collected at 0, 96, and 168 h of drought and after 96 h of recovery.

Fresh weight was measured immediately after harvesting the leaves. The dry weight was obtained by drying the same leaf material for several days in an oven at 80 °C until constant weight was measured. Leaf water content was calculated according to the formula WC = (FW − DW)/DW [51].

Samples for the biochemical analyses were frozen in liquid nitrogen and stored at −80 °C.

### 4.2. Biochemical Analyses

Approximately 250 mg of leaf material was grinded in 0.1% cold trichloroacetic acid (TCA) and was centrifuged for 30 min (15,000× *g*, 4 °C). The resulted supernatant was used for analyses of the stress markers.

Free proline was determined according to [52] with some modifications. Reaction mixture contained 0.5 mL supernatant, 0.5 mL 0.1% TCA, 1 mL conc. CH_3_COOH, and 1 mL ninhydrin reagent (1.25 g ninhydrin, 30 mL conc. CH_3_COOH, 20 mL 6M H_3_PO_4_). Supernatant was derivatized in the ninhydrin reagent for 1 h at 100 °C. After stopping the reaction in ice bath, the absorbance was read at 520 nm.

The level of biomembrane lipid peroxidation was assessed by the concentration of malondialdehyde in the plant tissues according to [53]. Five hundred microliters of supernatant was incubated with 1 mL 0.5% thiobarbituric acid in 20% TCA for 45 min at 100 °C. The absorbance of the resulting thiobarbituric reaction products was read at 532 nm and 600 nm. The content of MDA was calculated by using of 155 mM^−1^ cm^−1^ extinction coefficient. The hydrogen peroxide concentration was measured after incubation of 75 µL supernatant with 1 M KI (1:1 *v*/*v*) for 1 h in darkness [54]. The absorbance was read at 350 nm, and the concentration of H_2_O_2_ was calculated by a standard curve. The total content of the phenolic compounds was measured by following the method described by Swain and Goldstein [55] with some modifications. The reaction mixture consisted of 20 µL supernatant, 130 µL distilled H_2_O and 50 µL Folin–Ciocalteu reagent. After 3 min incubation 50 µL 1M Na_2_CO_3_ was added, and the reaction was left to develop for 2 h at room temperature. The absorbance was read at 725 nm, and the results were calculated by a standard curve prepared with known concentrations of gallic acid. The content of free thiol-groups-containing compounds was measured using Elman’s reagent [56]. The reaction mixture contained 40 µL supernatant and 150 µL Elman’s reagent. The absorbance was read at 412 nm after incubation for 10 min at room temperature.

For determination of the antioxidant enzyme activities approximately 200 mg leaf material was homogenized in cold 100 mM potassium phosphate buffer (K_2_HPO_4_/KH_2_PO_4_, pH 7.0, supplied with 1 mM EDTA) and 1% PVP. The homogenate was centrifuged at 15,000× *g* for 30 min at 4 °C. Catalase (EC 1.11.1.6) activity was measured by monitoring the degradation of H_2_O_2_ [57]. The reaction mixture consisted 50 µL supernatant, 2.930 mL reaction buffer (0.05M K_2_HPO_4_/KH_2_PO_4_, pH 7.0), and 20 µL 6% H_2_O_2_. The activity was measured by monitoring the degradation of H_2_O_2_ for 1 min at 412 nm. Guaiacol peroxidase (EC 1.11.1.7) activity was determined using guaiacol as electron donor. The reaction mixture consisted 20 µL supernatant, 1.1 mL reaction buffer (0.05M K_2_HPO_4_/KH_2_PO_4_, pH 7.0), 360 µL 1% guaiacol, and 20 µL 15% H_2_O_2_. The change of absorbance was followed at 470 nm [58]. The inhibition of the photochemical reduction of nitroblue tetrazolium was used to determine the activity of superoxide dismutase (EC 1.15.1.1). The amount of enzyme needed to cause a 50% inhibition was defined as one unit of SOD [59]. The activity of glutathione reductase was measured according to the method described by Smith et al. [60]. The reaction mixture consisted 100 µL supernatant, 1.180 mL reaction buffer (0.05M K_2_HPO_4_/KH_2_PO_4_, pH 7.5, 1 mM EDTA), 20 µL 50 mM DTNB, 0.1 mL 7.5 mM GSSG, 0.1 mL 1.5 mM NADPH. The reaction was monitored at 412 nm for 60 s. All enzyme activities were calculated on a protein basis. The total soluble protein content was measured according to Bradford [61].

The herbicide Serrate^®^ was purchased from Syngenta. All chemicals used for the biochemical analyses were purchased from Sigma. The measurements of the stress markers were conducted on Multiskan Spectrum spectrophotometer with microplate reader (Thermo Electron Corporation, Vantaa, Finland). The enzyme activities were measured on Shimadzu UV-1601 spectrophotometer (Shimadzu, Kyoto, Japan). A refrigerated Sigma 2-16K centrifuge (SciQuip, Wem, UK) was also used in the experiments.

### 4.3. Statistics

The experiments were repeated three times. The samples were collected in three replicates each. The data presented in the Figures are mean values ± SE. The significance of the treatments was assessed by one-way ANOVA with post-hoc Duncan’s multiple range test at *p* < 0.05.

## 5. Conclusions

The application of Serrate^®^ did not alter considerably biochemical, phenotypic, and growth traits of wheat plants during the experimental period. Drought stress substantially inhibited plant growth and provoked an increase in the studied biochemical parameters. We found that the stress markers, enzymatic and non-enzymatic antioxidant defense were additionally increased during the stress period after the combined herbicide+drought treatment. The recovery of the herbicide + drought treated plants was comparable to the one witnessed in the individuals subjected only to drought. It could be concluded that Serrate^®^ modulates the biochemical responses of wheat seedlings grown under drought stress but its action under adverse environment could not be explicitly characterized as cross-synergism or cross-adaptation without additional analyses.

## Data Availability

Not applicable.
